# Elimination of head and neck cancer initiating cells through targeting glucose regulated protein78 signaling

**DOI:** 10.1186/1476-4598-9-283

**Published:** 2010-10-27

**Authors:** Meng-Ju Wu, Chia-Ing Jan, Yeou-Guang Tsay, Yau-Hua Yu, Chih-Yang Huang, Shu-Chun Lin, Chung-Ji Liu, Yu-Syuan Chen, Jeng-Fan Lo, Cheng-Chia Yu

**Affiliations:** 1 Institute of Oral Biology, National Yang-Ming University, Taipei, Taiwan; 2 Department of Dentistry, National Yang-Ming University, Taipei, Taiwan; 3 Department of Pathology, China Medical University and Hospital, Taichung, Taiwan; 4 Institute of Biochemistry and Molecular Biology, National Yang-Ming University, Taipei, Taiwan; 5 Department of Dentistry, Taipei Veterans General Hospital, Taipei, Taiwan; 6 Graduate Institute of Chinese Medical Science and Institute of Medical Science, China Medical University, Taichung, Taiwan; 7 Institute of Basic Medical Science, China Medical University, Taichung, Taiwan; 8 Department of Health and Nutrition Biotechnology, Asia University, Taichung, Taiwan; 9 Institute of Oral Biology and Biomaterial Science, Chung Shan Medical University, Taichung, Taiwan; 10 Department of Dentistry, Chung Shan Medical University Hospital, Taichung, Taiwan

## Abstract

**Background:**

Head and neck squamous cell carcinoma (HNSCC) is a highly lethal cancer that contains cellular and functional heterogeneity. Previously, we enriched a subpopulation of highly tumorigenic head and neck cancer initiating cells (HN-CICs) from HNSCC. However, the molecular mechanisms by which to govern the characteristics of HN-CICs remain unclear. GRP78, a stress-inducible endoplasmic reticulum chaperone, has been reported to play a crucial role in the maintenance of embryonic stem cells, but the role of GRP78 in CICs has not been elucidated.

**Results:**

Initially, we recognized GRP78 as a putative candidate on mediating the stemness and tumorigenic properties of HN-CICs by differential systemic analyses. Subsequently, cells with GRP78 anchored at the plasma membrane (^mem^GRP78^+^) exerted cancer stemness properties of self-renewal, differentiation and radioresistance. Of note, xenotransplantation assay indicated merely 100 ^mem^GRP78^+ ^HNSCCs resulted in tumor growth. Moreover, knockdown of GRP78 significantly reduced the self-renewal ability, side population cells and expression of stemness genes, but inversely promoted cell differentiation and apoptosis in HN-CICs. Targeting GRP78 also lessened tumorigenicity of HN-CICs both *in vitro *and *in vivo*. Clinically, co-expression of GRP78 and Nanog predicted the worse survival prognosis of HNSCC patients by immunohistochemical analyses. Finally, depletion of GRP78 in HN-CICs induced the expression of Bax, Caspase 3, and PTEN.

**Conclusions:**

In summary, ^mem^GRP78 should be a novel surface marker for isolation of HN-CICs, and targeting GRP78 signaling might be a potential therapeutic strategy for HNSCC through eliminating HN-CICs.

## Background

Head and neck squamous cell carcinoma (HNSCC) ranks the third most common cancer in developing nations as well as the sixth worldwide [1]. In spite of improvements in the diagnosis and management of HNSCC, long-term survival rates have improved only marginally over the past decade [2]. Therefore, re-evaluating our current knowledge on HNSCC and developing novel therapeutic strategies is crucial. The reasonable explanation of this phenomenon is the existence of a rare subpopulation of cells within tumor that exhibit self-renewal capacity-the purported cancer stem cells (CSCs) or cancer initiating cells (CICs) [3,4]. CICs have been known to have the capacity to promote tumor regeneration and metastasis, and contribute to radio-resistance and chemo-resistance [5,6]. Experimental evidence for the existence of CICs has been reported for several tumor types, including brain, breast, colon, prostate, lung and HNSCC [7-12]. We previously demonstrated a subpopulation of HNSCCs displaying the characteristics of CICs using sphere formation assay [13]. However, the molecular characteristics and regulatory mechanisms that mediate HN-CICs properties remain unidentified. Therefore, uncovering key genes responsible for the maintenance of self-renewal and tumorigenicity in the HN-CICs is an imperative approach for new drug development.

GRP78/BiP/HSPA5, a central mediator of endoplasmic reticulum (ER) homeostasis, involves in the regulation of a variety of biological functions including protein folding, ER calcium binding, controlling of the activation of transmembrane ER stress sensors and cell survival [14]. Although the major subcellular localization of GRP78 is ER, GRP78 has been reported to be anchored at the plasma membrane [15]. It is well documented that GRP78 plays a crucial role in both stem cell and cancer biology. For instance, GRP78 is required for survival of embryonic stem cell precursors and is also highly expressed in hematopoietic stem cells [16]. Additionally, GRP78 is a mediator for tumor proliferation and metastasis, and confers resistance after chemotherapy and radiotherapy [15,17]. GRP78 is overexpressed in many tumor cells, including lung, breast, stomach, prostate, colon, and liver cancer [17,18]. In contrast, mice reducing GRP78 expression suppresses tumor development and promotes apoptosis [19]. Moreover, recent data point out that GRP78 regulates multiple malignant phenotypes of HNSCCs [20-22]. In addition, GRP78 is significantly up-regulated in breast disseminated tumor cells (DTC), which share the similar biological properties of CICs [23]. However, the role of GRP78 in CICs has never been determined. Based on these findings, it is worthy to investigate the importance of GRP78 in HNSCC tumorigenesis and in the maintenance cancer stemness properties of HN-CICs if GRP78 is preferentially overexpressed in CICs.

In the current study, we first identified GRP78/^mem^GRP78 expression was significantly increased in isolated HN-CICs, and ^mem^GRP78^+ ^cells posses higher tumorigenic potential and stemness properties. Consequently, we determined that a novel molecular pathway, GRP78 signaling, is linked to HN-CICs self-renewal and tumorigenicity. Overall, our studies provide evidence that inhibiting GRP78 signaling should be considered for further exploitation on therapeutic development for HNSCC.

## Results

### Elevation of GRP78 expression in Head and Neck Cancer Initiating Cells (HN-CICs)

**Figure 1:**
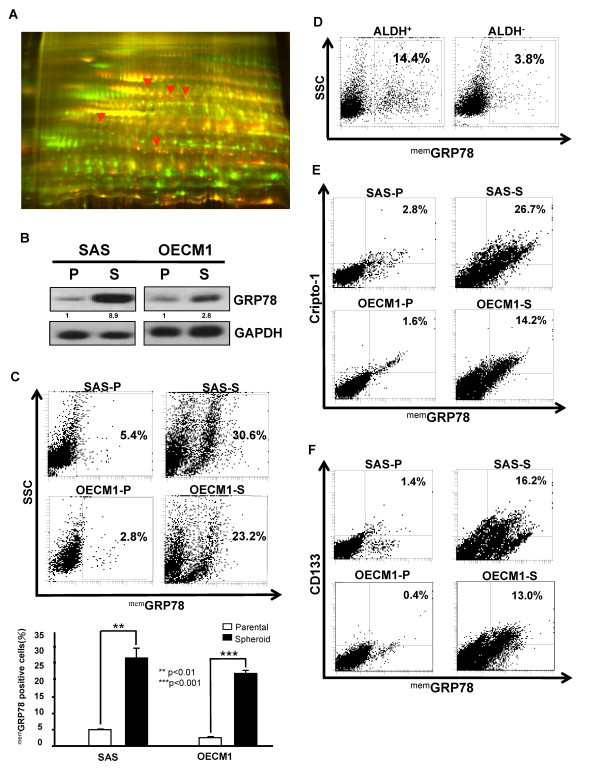
**The differential expression of GRP78 and membrane associated GRP78 (^mem^GRP78) in parental HNSCCs and HN-CICs**. (**A**) The whole cell proteomes of SAS cells (Cy3-labled, green) and SAS-derived sphere cells (HN-CICs) (Cy5-labeled, red) were collected and analyzed by two-dimensional differential gel electrophoresis (2-D DIGE). Image overlay of Cy3- and Cy5-labeled proteomes, red arrow indicates interests of up-regulated. (**B**) Total proteins were prepared from parental HNSCCs (SAS and OECM1) or HN-CICs (SAS and OECM1-derived spheres) and analyzed by immunoblotting with anti-GRP78 or anti-GAPDH antibodies as indicated. The amount of GAPDH protein of different crude cell extracts was referred as loading control. (**C**) ^mem^GRP78 positive cells expression in HNSCCs and HN-CICs was detected by FACS (**, p < 0.01; ***, p < 0.001). (**D**) The percentage of ^mem^GRP78 positive cells in isolated ALDH1^**+ **^and ALDH1^**- **^HNSCCs, respectively. The co-expression profile between ^mem^GRP78 and Cripto-1 (**E**) or CD133 (**F**) in HNSCCs and HN-CICs was examined by FACS. (P: Parental HNSCCs; S: HNSCCs-isolated sphere cells).

Previously, we have demonstrated the existence of HN-CICs [13]. To further elucidate the molecular mechanisms by which to mediate the self-renewal ability and tumorigenicity of HN-CICs, molecular targets specifically expressed in HN-CICs were to be identified. The differential expression profile between HN-CICs and HNSCCs was examined by either systemic transciptome analysis or two-dimensional differential gel electrophoresis (2-D DIGE) followed by mass spectroscopy analysis. We noticed that the transcripts and protein level of GRP78 were significantly up-regulated in enriched HN-CICs (Additional file 1 and Figure 1A). To further validate the results from Affymatrix microarray and proteomic analyses, western blotting was performed. Immunoblotting analyses showed that antibody against GRP78 detected more GRP78 protein in crude cell extracts of enriched HN-CICs than in that of parental HNSCCs (Figure 1B).

Recent findings of GRP78 on plasma membrane of cancer cells but not on normal cells suggest that targeted therapy against surface GRP78 of cancer cells may be effective [24]. Compared to parental HNSCCs, we found more membrane-associated GRP78 positive (^mem^GRP78^+^) cells in HN-CICs by FACS analyses (Figure 1C). In addition, it has been demonstrated that aldehyde dehydrogenase 1 (ALDH1) activity could be used as a selection marker to isolated breast cancer CICs and head and neck CICs [25,26]. Consistent with tumor spheres formation ability, ALDH1^+ ^HNSCCs also displayed more ^mem^GRP78^+ ^cells (Figure 1D). Finally, HN-CICs showed elevated co-expression of either CD133 or Cripto-1 with ^mem^GRP78 in comparison to parental HNSCCs (Figure 1E and 1F), where both CD133 and Cripto-1, the well known CICs markers, have been used to identify CICs [13,27,28]. Taken together, we hypothesized that up-regulation of GRP78/^mem^GRP78 is pivotal for maintenance cancer stemness characteristics of HN-CICs.

### ^mem^GRP78^+ ^HNSCCs display cancer initiating cells properties *in vitro *and *in vivo*

**Figure 2:**
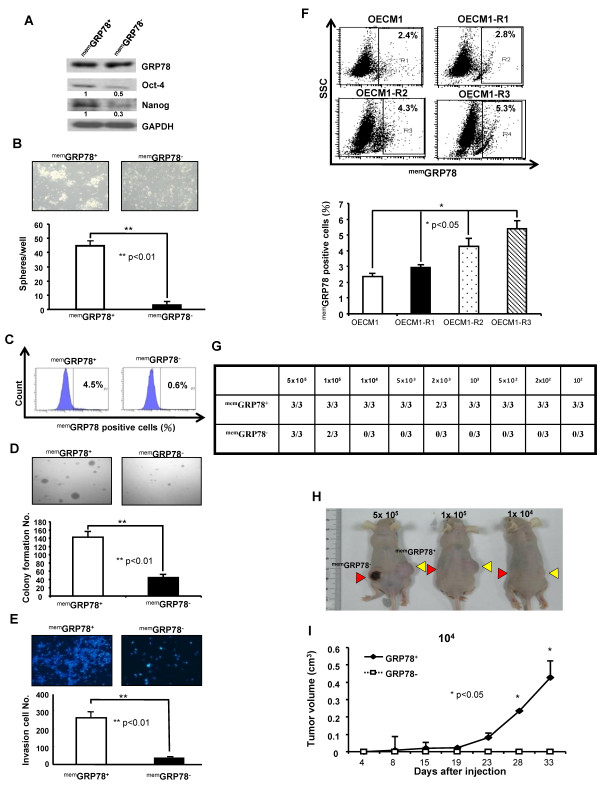
**Cancer stem cells properties of ^mem^GRP78^+ ^and ^mem^GRP78^- ^HNSCCs *in vitro *and *in vivo***. **(A) **Expressions of pluripotent stemness genes (Oct4 and Nanog) in ^mem^GRP78^+ ^and ^mem^GRP78^- ^HNSCCs were determined by western analysis. The amount of GAPDH was referred as loading control. **(B) **Representative images of tumorsphere-forming ability in ^mem^GRP78^+ ^and ^mem^GRP78^- ^HNSCCs grown under defined serum-free selection medium as described at Materials and Methods. The numbers of spheres were further calculated using microscope. Results are means ± SD from three experiments. (**, p < 0.01) **(C) **Sorted ^mem^GRP78^+ ^and ^mem^GRP78^- ^cells were further cultivated with standard medium containing 10% serum. At day 10, the percentage of ^mem^GRP78 expression was re-analyzed by flow cytometry. **(D) **To elucidate the anchorage independent growth, single cells suspension of ^mem^GRP78^+ ^and ^mem^GRP78^- ^cells plated onto soft agar and analyzed. Results are means ± SD of triplicate samples from three experiments (**, p < 0.01) **(E) **Invasion ability of ^mem^GRP78^+ ^and ^mem^GRP78^- ^cells were plated onto transwell coated with matrigel and analyzed. Results are means ± SD of triplicate samples from three experiments (**, p < 0.01) **(F) **Increased radio-resistance properties (OECM1-R3 > OECM1-R2 > OECM1-R1 > parental OECM1) positively correlates ^mem^GRP78 expression in HNSCCs by FACS analysis. (*, p < 0.05) **(G) **Summary of the *in vivo *tumor growth ability of different numbers of ^mem^GRP78^+ ^and ^mem^GRP78^- ^cells examined by xenotransplantation analysis. **(H) **Representative tumor growth of ^mem^GRP78^+ ^and ^mem^GRP78^- ^HNSCCs was generated in the subcutaneous space of recipient nude mice (Yellow arrows: ^mem^GRP78^+ ^HNSCCs; Red arrows: ^mem^GRP78^- ^HNSCCs). **(I) **Tumor volume was measured after inoculation of ^mem^GRP78^+ ^and ^mem^GRP78^- ^HNSCCs in nude mice. Error bars correspond to SD (*lower panel*). (*, p < 0.05).

To test whether ^mem^GRP78^+ ^HNSCCs had the CICs characteristics, SAS cells were sorted into ^mem^GRP78^+ ^and ^mem^GRP78^- ^cells by flow cytometry (Additional file 2A). Compared with ^mem^GRP78^- ^SAS cells, the ^mem^GRP78^+ ^SAS cells displayed higher levels of protein and mRNA of stemness genes (Oct-4 and Nanog) (Figure 2A and Additional file 2B). We next performed tumor spheres assay for evaluating the self-renewal ability of ^mem^GRP78^+ ^and ^mem^GRP78^- ^cells, respectively. Interestingly, ^mem^GRP78^+ ^cells had higher tumor spheres-forming capability than ^mem^GRP78^- ^HNSCCs (Figure 2B). When isolated ^mem^GRP78^+ ^and ^mem^GRP78^-^cells were first cultivated within 10% serum for 10 days, then the cell surface GRP78 expression profile was further analyzed by flow cytometry, respectively. We observed that ^mem^GRP78^+ ^cells regenerated both ^mem^GRP78^+ ^and ^mem^GRP78^- ^cells, whereas, ^mem^GRP78^+ ^cells were not detectable from cultivated ^mem^GRP78^- ^cells (Figure 2C). These data indicate that ^mem^GRP78^+ ^HNSCCs could re-differentiate into ^mem^GRP78^- ^cells. To address whether the tumorigenic activity differed between ^mem^GRP78^+ ^and ^mem^GRP78^- ^cells, *in vitro *tumorigenic properties including matrigel invasion and anchorage independent growth, and *in vivo *xenografts assay were performed. The colony/invasion formation abilities of ^mem^GRP78^+ ^HNSCCs were significantly higher than those of the ^mem^GRP78^- ^HNSCCs (Figure 2D and 2E). To further evaluate the correlation between ^mem^GRP78 expression profile and radioresistance, we established radioresistant (R) HNSCCs (R1, R2, and R3) by serially fractionated irradiation (see details from Material and methods). We found that the expression profile of ^mem^GRP78 was significantly enhanced in radioresistant HNSCCs (Figure 2F; R3>R2>R1>Parental OECM1). For *in vivo *xenotransplantation assay, we observed that 10000 GRP78^- ^cells did not induce tumor formation but 100 GRP78^+ ^HNSCCs resulted in the generation of visible tumors 4 weeks after injection in xenotransplanted mice (Figure 2G, H, and 2I, Additional file 2D, 2E and 2F). Collectively, ^mem^GRP78 positive cells possess the capabilities for self-renewal, differentiation, radioresistance and high *in vivo *tumorigenicity.

### Down-regulation of GRP78 reduces self-renwal properties and inhibits tumorigenicity of HN-CICs

**Figure 3:**
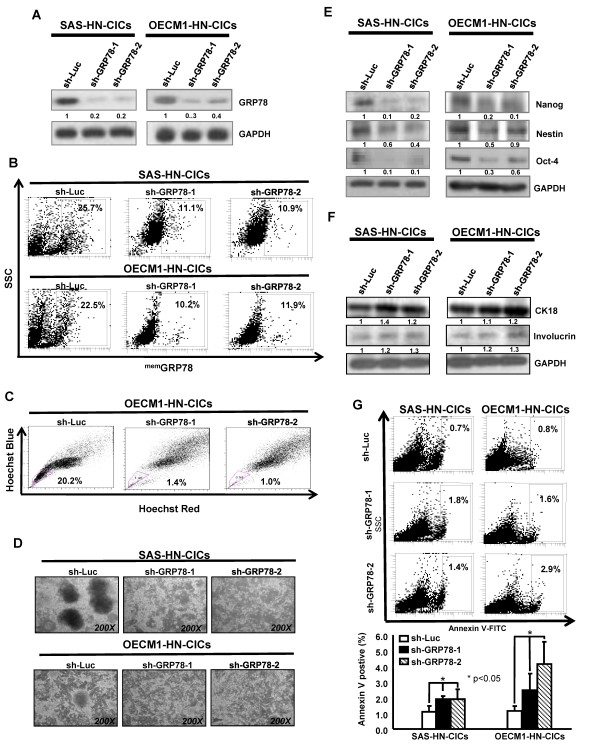
**Suppression of GRP78/^mem^GRP78 expression diminished spheres-forming capability, stemness genes expression, and side population cells of HN-CICs**. **(A) **Down-regulation of GRP78 in HN-CICs (SAS (*left panel*) and OECM1 (*right panel*) mediated by shRNAi was validated by western blotting) **(B) **The percentages of ^mem^GRP78^+ ^cells in sh-GRP78 knockdown and sh-Luc HN-CICs were compared by flow cytometry analysis, respectively. **(C) **Single cell suspensions of sh-GRP78 and sh-Luc-expressing HN-CICs incubated with Hoechst 33342 were examined for side population by flow cytometry. **(D) **HNSCCs-enriched sphere cells were first infected with Sh-GRP78-1, Sh-GRP78-2 or Sh-Luc lentivirus, and further cultivated under the serum-free defined selection medium. The tumor sphere formation capability and cellular morphology of enriched HN-CICs (*Upper panel*, SAS; *Lower panel*; OECM1) treated with either sh-Luc or GRP78*-*shRNA lentivirus were examined with microscope. **(E) **Total proteins from figure 3d were isolated and immublotted by using antibodies against, anti-Oct-4, anti-Nanog, anti-Nestin or anti-GAPDH antibodies as indicated. The amount of GAPDH protein of different crude cell extracts was referred as loading control. **(F) **Protein level of epithelial specific differentiation markers, CK18 and invoclurin in enriched HN-CICs cells infected with sh-Luc, or sh-GRP78 lentivirus was assessed by western blot. **(G) **Single cell suspension of spheres prepared from figure 3d transduced with sh-Luc or sh-GRP78 lentivirus were stained with Annexin V and examined by flow cytometry. The experiments were repeated three times and representative results were shown. Results are means ± SD (*, p < 0.05).

To further investigate the crucial role of GRP78 up-regulation in maintaining biological properties of HN-CICs and HNSCCs, we performed the loss-of-function approach to evaluate the effect of GRP78 knockdown on HNSCCs derived HN-CICs. First, the HNSCCs derived HN-CICs were generated by cultivating HNSCCs under defined serum-free medium as described [13]. Then, the enriched HN-CICs were infected with lentivirus expressing either small hairpin RNA (shRNA) targeting GRP78 (shGRP78) or shRNA against luciferase (shLuc), respectively. HN-CICs infected with shLuc lentivirus were used as control cells. Successful infected HN-CICs was validated as the Green Fluorescence Protein (GFP) positive cells since GFP was co-expressed as a reporter marker for cell transduction (data not shown). Western blot analyses confirmed that both sh-GRP78*-1 *and sh-GRP78*-2 *markedly repressed GRP78 protein expression in both HN-CICs and HNSCCs (Figure 3A and Additional file 3A). ^mem^GRP78^+ ^cells were also reduced in shGRP78-expressing HN-CICs and HNSCCs (Figure 3B and Additional file 3B). Differential levels of GRP78 suppression between membrane and cytosol in head and neck cancer initiating cells by western blotting and flow cytometry results were examined in Additional file 3C.

Tumor-derived side population (SP) cells also have been found to have characteristics of cancer stemness [29].GRP78 depletion significantly decreased the side population in HN-CICs and HNSCCs, respectively (Figure 3C and Additional file 3D). To further investigate whether GRP78 expression plays a role in maintaining self-renewal or cancer stem-like properties in HN-CICs directly, the HNSCCs-derived tumor spheres, afterward transduction with Sh-GRP78 lentivirus, did not maintain floating spheres but show more attached epithelial-like cells (Figure 3D). In opposite, HN-CICs after Sh-GRP78 lentiviruses infection displayed decreased expression of "cancer stemness" genes (Oct-4, Nanog, and Nestin) but enhanced expression of epithelial differentiation marker, CK18 and Involucrin (Figure 3E and 3F). To determine whether the reduction in tumor sphere formation efficiency with GRP78 down-regulation is due to decreased HN-CICs survival, we determined the percentage of apoptotic cells using Annexin V staining. HN-CICs transduced with Sh-GRP78 lentivirus significantly increased the percentage of Annexin V-positive cells (Figure 3G). Together, these results further support that the loss of GRP78 resulted in a decrease of CICs properties due to up-regulation differentiation and apoptotic activity.

**Figure 4:**
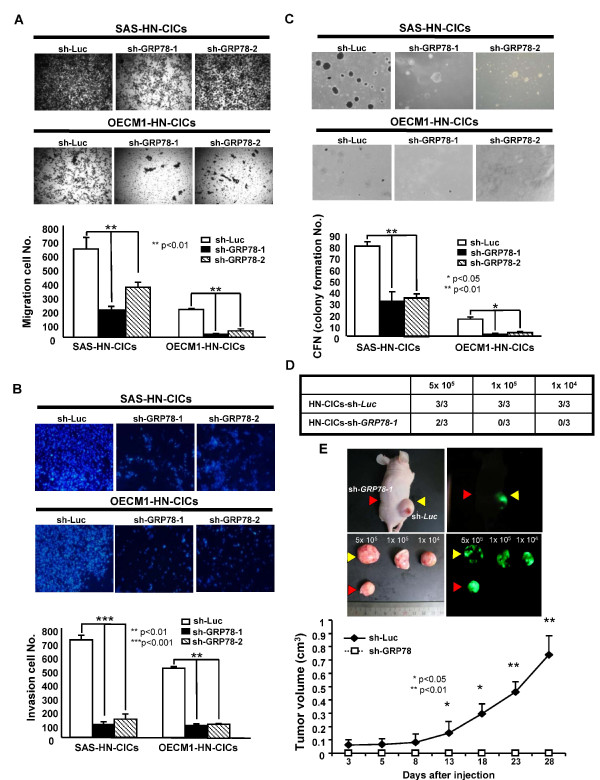
**GRP78 inhibition impaired *in vitro *and *in vivo *tumorigenic properties of HN-CICs**. **(A) **To elucidate the capability of migration of GRP78 shRNA knockdown and sh-Luc HN-CICs, single cell suspension of GRP78-specific shRNA or control sh-Luc HN-CICs were plated onto transwell and analyzed as described in Materials and Methods. Results are means ± SD of triplicate samples from three experiments (**, p < 0.01). (**B) **Single cell suspension of GRP78-specific shRNA or control sh-Luc HN-CICs were plated onto transwell coated with matrigel and analyzed as described in Materials and Methods. Data are means ± SD of triplicate samples from three experiments (**, p < 0.01; ***, p < 0.001). (**C) **To elucidate the anchorage independent growth, single cell suspension of stable GRP78-specific shRNA or control sh-Luc HN-CICs (SAS (*upper panel*), OECM1 (*lower panel*)) were plated onto soft agar and analyzed as described in Materials and Methods. Results are means ± SD of triplicate samples from three experiments (*, p < 0.05; **, p < 0.01). **(D) **Summary of the *in vivo *tumor growth ability of different numbers of GRP78-knockdown or control (sh-Luc) HN-CICs examined by xenotransplantation analysis. **(E) **Representative tumor growth of 10000 control and 10000 GRP78-knockdown HN-CICs was generated in the subcutaneous space of recipient mice (*upper panels*).Tumor volume was measured after inoculation of GRP78-knockdown shRNA and sh-Luc-expressing HN-CICs (Yellow arrows: sh-Luc-expressing HNSCCs; Red arrows: sh-GRP78-expressing HNSCCs) (*lower panel*). Error bars correspond to SD (*, p < 0.05; **, p < 0.01).

To elucidate the direct effect of GRP78 knockdown on *in vitro *tumorigenic properties including cell migration, matrigel invasion and anchorage independent growth of HN-CICs, single cell suspension of control- or GRP78*-*knockdown HN-CICs were plated onto transwell chamber (Figure 4A), onto transwell chamber coated with matrigel (Figure 4B) or into soft agar (Figure 4C), and analyzed as described in Materials and Methods, respectively. The migratory/invasion/colony formation abilities of GRP78 knockdown HN-CICs were significantly reduced than those of the control HN-CICs (Figure 4A, B, and 4C). We next sought to determine if down-regulation of GRP78 expression could attenuate the tumor initiating activity of HN-CICs *in vivo*. Strikingly, GRP78-knockdown HN-CICs gave rise to a new tumor at 5x10^5 ^in one of six mice, however, HN-CICs control cells generated tumor when 1x10^4 ^cells were injected into nude mice (three out of three mice)(Figure 4D). In addition, knockdown of GRP78 expression in HN-CICs and HNSCCs significantly reduced the tumor volumes (Figure 4E and Additional file 3E). Overall, our data indicate that down-regulation of GRP78 inhibited *in vitro *tumorigenicity and *in vivo *tumor-initiating activity of HN-CICs.

### Overexpression of GRP78 in HNSSCs enhances in vitro malignant potentials and ^mem^GRP78^+ ^expression profile

To evaluate whether overexpression of GRP78 could enhance tumorigenic properties of HNSCCs, we generated HNSCCs with transient overexpression of GRP78 by transfection with plasmids overexpressing GRP78 protein into HNSCCs. Total proteins from 293T cells or HNSCCs (SAS) with transfection of GRP78 expressing plasmids displayed elevated expression of GRP78 (Additional file 4A). Furthermore, we demonstrated that GRP78 overexpression also resulted in increased ability on *in vitro *cell migration (Additional file 4B). To evaluate whether overexpressios of GRP78 on promoting ^mem^GRP78^+ ^cells in HNSCC, SAS cells were co-transfected with plasmids expressing green fluorescence protein (GFP) and GRP78. We discovered GFP positive cells (meaning cells under successful transfection) showed more ^mem^GRP78^+ ^in co-transfected cells than control cells (Additional file 4B). Together, our data demonstrated that overexpression of GRP78 not only enhanced in vitro malignancy but also expression profile of ^mem^GRP78^+ ^in HNSCCs.

### Co-expression of GRP78 and Nanog in HNSCC tissues

**Figure 5:**
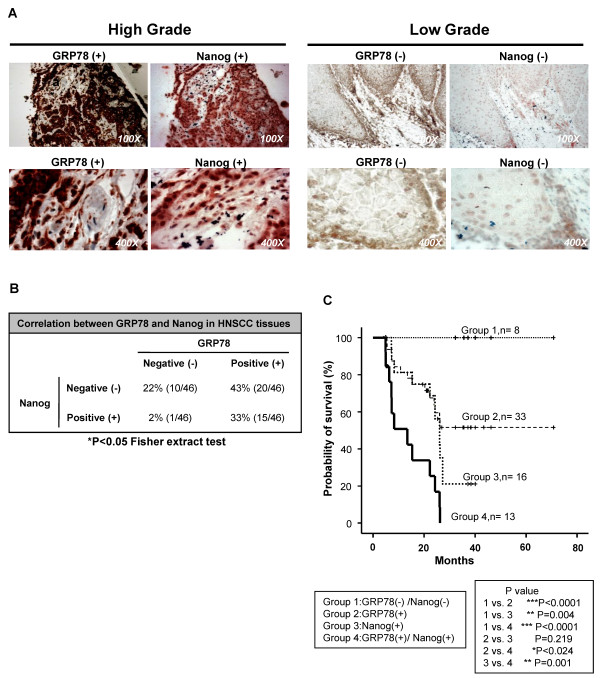
**Co-expression of GRP78 and Nanog in HNSCC tissues**. **(A) **Representative pictures of double positive (*left panel*) and double negative (*right panel*) in 46 HNSCC patient cases. Magnification was shown at lower right corner. **(B) **Statistical analysis of correlation betweenGRP78 and Nanog by Fisher extraction text. **(C) **Kaplan-Meier analysis of overall survival in HNSCC patients according to the expression of GRP78 and Nanog (Group1: GRP78(+)Nanog(+), Group2: GRP78(+), Group3: Nanog (+) and Group4: GRP78 (-)Nanog(-)).(*, p < 0.05; **, p < 0.01; ***, p < 0.001).

We have been reported that HNSCC patients with abundant Nanog protein expression are more likely to have poor survival outcomes [13]. Overexpression of GRP78 also correlates with poor HNSCC prognosis [30]. To further investigate the correlation between GRP78 and Nanog levels in human cancers, we established the ontogeny of GRP78 and Nanog co-expression by tissue immunohistochemical staining with a panel of specimens array of 46 HNSCC patients. Two representative cases with double-positive or double-negative of GRP78 and Nanog were shown in Figure 5A. We found co-expression of GRP78 and Nanog in the moderate to poor-differentiated HNSCC tissues rather than in well-differentiated HNSCC tissues (Figure 5A). The significant correlation between the expression of GRP78 and Nanog in HNSCC tissues was determined (Figure 5B, p < 0.05). To investigate the prognostic significance of the expression GRP78 and Nanog patterns in HNSCC, we divided patients into four groups: GRP78 (+)Nanog (+), GRP78 (+), Nanog (+), and GRP78 (-)Nanog(-) HNSCC patients. The Kaplan-Meier analyses showed that co-expression of GRP78 and Nanog predicted the worse overall survival than all other HNSCC patients (Figure 5C).

### GRP78 knockdown promotes apoptosis via survival signaling in HN-CICs

**Figure 6:**
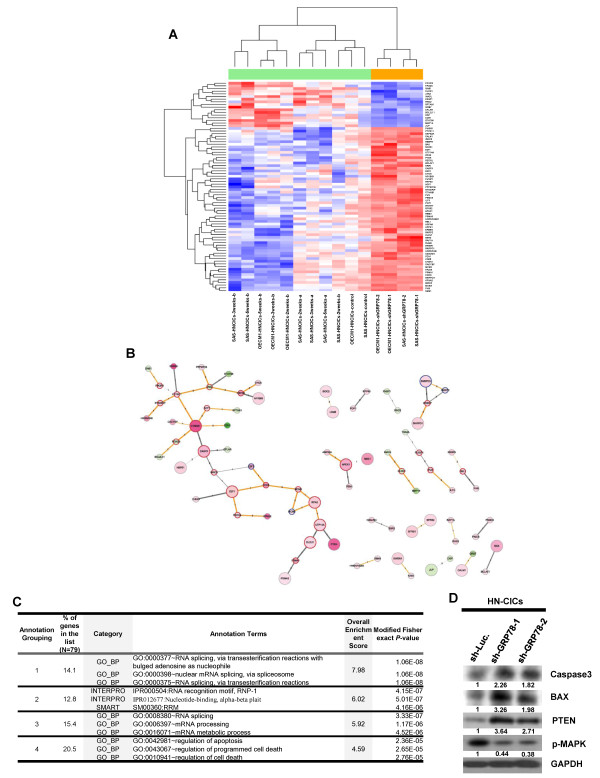
**Differentially expressed genes in GRP78-knockdown HN-CICs**. **(A) **A total of 79 significantly differentially expressed genes mapped in the human PPIs were clustered (by row) according to their similarities in GRP78*-*knockdown HN-CICs, red indicating induction and blue indicating repression. **(B) **Mapped human PPIs among the differentially expressed genes were grouped according to the topological characteristics as highlighted in border colors (periphery: gray; inter-modular hubs: red; and intra-modular hubs: blue). Color legends were according to expression patterns: as for nodes, red - induction and green - repression; as for edges, gray -negatively correlated and orange-positively correlated. Thickness of edges was proportional to the absolute value of PCC and numbers indicated databases reporting such interactions. **(C) **Top 4 functional annotation clusters analyzed from DAVID were listed. **(D) **Total proteins were prepared from Sh-Luc and Sh-GRP78 expressing HN-CICs and analyzed by immunoblotting with antibodies against Caspase-3, BAX, PTEN, MAPK or GAPDH as indicated. The amount of GAPDH protein of different crude cell extracts was referred as loading control.

To identify the systemic differential gene expression profile by down-regulation of GRP78 in HN-CICs, we performed Affymetrix microarray analyses. Upon the knockdown of GRP78, we identified 434 probes consistently induced or repressed and mapped them onto the human PPIs. We filtered the mapped PPIs among the differentially expressed genes by their co-expression of the reactants in the GRP78-knockdown HN-CICs (PCCs > 0.5). As shown in Figure 6A and 6B, 79 genes and 64 interactions were retained in the final networks. The direction and strength of co-expression were depicted in Figure 6B. Highly correlated genes were CTNNB1 v.s. PTPN11, E2F1 v.s. CDC6, E2F1 v.s. RECQL, and MCM5 v.s. RPA2, with positive PCCs, as well as CHEK1 v.s. E2F1, PSMA1 v.s. DLEU1, and HSPA8 v.s. NFKBIB, with negative PCCs. Topologically, 24 inter-modular hubs, 4 intra-modular hubs, and 51 periphery genes. Functional annotation of the 79 genes in the networks of GRP78 knockdown in HN-CICs was summarized in Figure 6C. To further study the possible mechanisms involved in GRP78-mediated cancer stemness properties, we found out knockdown of GRP78 enhanced the expression of PTEN, BAX and Caspase3 but reduced the expression of p-MAPK in HN-CICs (Figure 6D). These results support PTEN-PI3K-Akt and ERK signaling is regard as crucial pathways in mediating CICs characteristics [31,32]. Additionally, GRP78 might regulate survival pathways to modulate HN-CICs behaviors.

## Discussion

The emerging importance of the stress response and molecular chaperones in stem cells oncogenesis is well recognized [33,34]. However, the relationship between a stress-inducible endoplasmic reticulum chaperone and cancer stem cells remains unclear. In this current study, we first identified GRP78/^mem^GRP78, a stress-inducible endoplasmic reticulum (ER) chaperone, was significantly elevated in isolated HN-CICs through two-dimensional differential gel electrophoresis or transcriptome profiling analysis (Figure 1A and Additional file 1). Consequently, GRP78^+ ^HNSCCs cells displayed CICs properties in comparison to ^mem^GRP78^- ^compartments (Figure 2). We thus directly evaluated the functional role of GRP78 in the maintenance of stemness characteristics and tumorigenic phenotype of HN-CICs. Lentiviral shRNA-mediated knockdown of GRP78/^mem^GRP78 decreased self-renewal ability, side population cells, stemness genes expression in HN-CICs (Figure 3). Furthermore, analysis of the cell survival and differentiation ability of shGRP78-HN-CICs revealed that loss of GRP78 directly caused a decrease of the CICs subpopulation due to increasing of apoptotic and differentiated cells (Figure 3F and 3G). These results indicate that GRP78 directly contributes to the self-renewal and survival of HN-CICs. Increased tumorigenic activity is key hallmark of HN-CICs, strikingly; we also found that knockdown of GRP78 lessened tumor initiating activity of HN-CICs both *in vitro *and *in vivo *(Figure 4). These results suggest that elevated GRP78 signaling is associated with stemness propeties and tumorigenic potentials of HNSCCs.

It has been reported that GRP78 signaling is crucial for cell survival/apoptosis via various apoptotic signaling pathways [35,36]. In the ER membrane, GRP78 interacts with caspase 7 and formed an antiapoptotic complex [37]. Additionally, GRP78 it has been shown that GRP78 represses the activation of Bax and the release of cytochrome C from the mitochondria. Overexpression of GRP78 in glioblatomas cells renders these cells resistant to etoposide- and cisplatin- induced apoptosis [38]. In contrast, knockdown of GRP78 decreases cell proliferation and sensitizes glioma cells to chemoradiotherapy through the activation of caspase 7 cleavage [38]. GRP78 has also been implicated in proliferation properties through activation of the Akt pathways [39,40]. Recently, knockdown GRP78 or Cripto disrupts of the Cripto binding to cell surface GRP78 in cancer cells inhibits oncogenic signaling via MAPK/PI3K and Smad2/3 pathways [41]. In accordance with other findings, silencing of GRP78 increased BAX and Caspase3 but reduced the expression of p-MAPK in head and neck cancer initiating cells (Figure 6). Collectively, our data first demonstrated the crucial role of GRP78 in the proliferation/apoptosis property of head and neck cancer initiating cells.

Low oxygen tension or hypoxic condition plays an important role in both the developing embryo and the adult as specific niches [42]. Hypoxia is also a common microenvironmental factor/niche that adversely influences tumor aggressiveness and treatment response [43]. Recently, many reports demonstrated hypoxia is also crucial in maintaining the stem cells and CICs niche. For example, hypoxia increases SP cells having high tumorigenicity and CICs characteristics including Oct-4 up-regulation [44]. We also observed that HIF-1α was up-regulated in our enriched HN-CICs (data not shown). However, the *hypoxia*-inducible factors (HIFs) function through the transcriptional regulation of a number of important gene products [45]. Notably, it is evident that HIF1a and HIF2a can often play non-overlapping biological roles due to their unique target genes. HIF-1a promotes CD133-positive human glioma-derived CICs propagation and self-renwal [46,47]. Whereas, HIF-2a is an important primary regulator of hypoxic responses, which shows strong tumor-promoting activity and has been shown to bind to the Oct-4 promoter and induce Oct-4 expression in ES cells [48]. Cellular adaptation to hypoxia occurs through multiple mechanisms, including activation of the unfolded protein response (UPR) in which GRP78 plays a crucial role [49,50]. Ostergaard and colleagues reveal that lowering O_2_, probably in part through HIF, may upregulate the expression of GRP78 [51]. Additionally, the elevation of GRP78/^mem^GRP78 was also observed in HIF1a or HIF2a-overexpressing HNSCCs (data not shown). Previously, we observed that enhanced expression of Oct-4, Nanog and CD133 in our isolated HN-CICs [13]. Moreover, lentiviral knockdown of GRP78 expression decreased stemness properties in HN-CICs. Based on these findings, we proposed that HIF-mediated up-regulation of GRP78 might provide HN-CICs with stemness and tumorigenic properties.

In addition, Arnaudeau et al have demonstrated that GRP78 directly interacts with P53 for stabilization and inactivation in trophoblast and nasopharyngeal carcinoma [52]. Lin et al report that P53 negatively regulates the transcriptional activity of stem cell marker, Nanog [53]. We also found that downregution of GRP78 reduced the Nanog expression in HN-CICs (Figure 3E). Therefore, our current hypothesis is that the interaction between GRP78 and p53 abrogates the negative regulation of p53 on Nanog. However, future research delineating the details of how GRP78 regulates its downstream targets and how these interactions influence the stemness properties of CICs remain to be determined.

Increased tumor initiating activity is hallmark of CSCs [12]. Knockdown of GRP78 lessened tumor initiating activity both *in vitro *and *in vivo*. However, deletion of GRP78 did not completely eliminate and CICs properties tumor initiation potential of HN-CICs (Figure 4D). It is reasonable that GRP78 signaling may not be the only one pathway in contributing in the regulation of HN-CICs, although, we and others observed that GRP78 regulates Wnt5A and PTEN-PI3K-Akt expression [54]. Other developmental signaling pathways, including Notch, Hedgehog signaling and Bmi1 signaling have been reported to play critical roles in the regulation of various CICs characteristics, which were not significant changed in GRP78-knockdown HN-CICs. Abnormal functions and regulations of components of these signaling pathways are often associated with different cancers, implicating potential roles of these signaling pathways in the CICs derived from different tissue origin. It would be interesting to determine the potential cross-linking of GRP78 signaling with other signaling pathways. These studies also suggest that the use of a combination of inhibitors for multiple signaling pathways might be more effective than blockade of single pathway regulating HN-CICs.

## Conclusions

Together, our present research shows that a novel pathway, GRP78 signaling, plays a major role in the maintenance of HN-CICs population. Targeting GRP78 signaling might be a potential therapeutic target for HNSCC by eliminating HN-CICs. In addition, co-expression of GRP78 and Nanog should be useful prognostic factors for HNSCC patients.

## Materials and methods

### Cell lines cultivation and enrichment of HN-CICs from HNSCCs

Originally, SAS was grown in DMEM, and OECM1 was grown in RPMI supplemented with 10% fetal bovine serum (FBS) (Grand Island, NY), respectively. The two cell lines were then cultured in tumor sphere medium consisting of serum-free DMEM/F12 medium (GIBCO), N2 supplement (GIBCO), 10 ng/mL human recombinant basic fibroblast growth factor-basic (FGF) and 10 ng/mL Epidermal Growth Factor (EGF) (R&D Systems, Minneapolis, MN) (. Cells were plated at a density of 7.5 × 10^4 ^live cells/10-mm dish, and the medium was changed every other day until the tumor sphere formation was observed in about 4 weeks [13].

### RNA Isolation and Affymetrix GeneChip Analysis

RNA wasextracted from cells using Trizol reagent (InvitrogenLife Technologies), purity confirmed by OD260:280 ratio and analyzed using formaldehyde gel electrophoresis. For Affymetrix GeneChipanalysis, RNAeasy kit (Qiagen, Valencia, CA) was used forfurther RNA purification. Gene profiling was performedusing Affymetrix Human Genome U133 plus 2.0 (containing 47,000 transcripts and variants, including 38,500 well-characterized human genes) for the microarrays hybridization at the genomic core facilities at the National Yang-Ming University Genome Research Center.

### Construction of Lentiviral-mediated RNAi for silencing GRP78

The pLV-RNAi vector was purchased from Biosettia Inc. (Biosettia, San Diego, CA). The method of cloning the double-stranded shRNA sequence is described in the manufacturer's protocol. Lentiviral vectors expressing short hairpin RNA (shRNA) that targets human GRP78 (oligonucleotide sequence: Sh-GRP78-1:5'- AAAAGCCTAAATGTTATGAGGATCATTGGATCCAATGATCCTCATAACATTTAGGC -3';Sh-GRP78-2:5'-AAAAGGAGCGCAUUGAUACUAGATTTTGGATCCAAAATCTAGTATCAATGCGCTCC-3') were synthesized and cloned into pLVRNAi to generate a lentiviral expression vector. Lentivirus production was performed by transfection of plasmid DNA mixture with lentivector plus helper plasmids (VSVG and Gag-Pol) into 293T cells using Lipofectamine 2000 (LF2000, Invitrogen, Calsbad). Supernatants were collected 48 hours after transfection and then were filtered; the viral titers were then determined by FACS at 48 hours post-transduction. Subconfluent cells were infected with lentivirus in the presence of 8 μg/ml polybrene (Sigma-Aldrich). The GFP is expressed in lentivirus-infected cells as the marker to indicate that the cells express the shRNA for silencing GRP78.

### Aldefluor assay and flow cytometry

To measure and isolate cells with ALDH activity, the Aldefluor assay was performed according to manufacturer's (Stemcell Technologies, Durham, NC, USA) guidelines. Dissociated single cells were suspended in Aldefluor assay buffer containing the ALDH substrate, Bodipy-aminoacetaldehyde (BAAA) at 1.5 mM and incubated for 40 min at 37°C. To distinguish between ALDH-positive and ALDH-negative cells, a fraction of cells was incubated under identical condition in the presence of a 10-fold molar excess of the ALDH inhibitor, diethylaminobenzaldehyde (DEAB). This results in a significant decrease in the fluorescence intensity of ALDH-positive cells and was used to compensate the flow cytometer.

### Side population analysis

Cells were resuspended at 1 × 10^6^/mL in pre-warmed DMEM with 2% FCS. Hoechst 33342 dye was added at a final concentration of 5 μg/mL in the presence or absence of verapmil (50 μM; Sigma) and was incubated at 37°C for 90 min with intermittent shaking. At the end of the incubation, the cells were washed with ice-cold HBSS with 2% FCS and centrifuged down at 4°C, and resuspended in ice-cold HBSS containing 2% FCS. Propidium iodide at a final concentration of 2 μg/mL was added to the cells to gate viable cells. The cells were filtered through a 40-μm cell strainer to obtain single cell suspension before analysis. The Hoechst 33342 dye was excited at 357 nm and its fluorescence was dual-wavelength analyzed (blue, 402-446 nm; red, 650-670 nm). Analyses were done on FACSAria (BD, San Diego, CA).

### Establish radiation resistant cell line

Cells were seeded on 75T flask at a density of 2 × 10^5 ^in medium; kept culturing part of the cells for next radiation treatment after ionizing irradiation and repeat three times. The radiation resistant (R1, R2 and R3) cells were for further experiments. The g-radiation (ionizing irradiation) was delivered by Theratronic cobalt unit T-1000 (Theratronic International) at a dose rate of 1.1 Gy/min (SSD = 57.5 cm).

### *In vitro *cell migration Assay

For transwell migration assays, 2 × 10^5 ^cells were plated into the top chamber of a transwell (Corning, Acton, MA) with a porous transparent polyethylene terephthalate membrane (8.0 μm pore size). Cells were plated in medium with lower serum (0.5% FBS), and medium supplemented with higher serum (10% FBS) was used as a chemoattractant in the lower chamber. The cells were incubated for 24 h and cells that did not migrate through the pores were removed by a cotton swab. Cells on the lower surface of the membrane were stained with Hoechst 33258 (Sigma-Aldrich) to show the nuclei; fluorescence was detected at a magnification of 100× using a fluorescence microscope (Carl Zeiss, Oberkochen, Germany). The number of fluorescent cells in a total of five randomly selected fields was counted.

### *In vitro *cell invasion analysis

The 24-well plate Transwell^®^ system with a polycarbonate filter membrane of 8-μm pore size (Corning, United Kingdom) was employed to evaluate the invasion ability of cells. The membrane was coated with Matrigel™ (BD Pharmingen, NJ, USA). The cancer cell suspensions were seeded to the upper compartment of the Transwell chamber at the cell density of 1 × 10^5 ^in 100 μl within serum-free medium. The lower chamber was filled with media with 10% serum. After 24 hours of incubation, the medium was removed and the filter membrane was fixed with 4% formalin for 1 hour. Subsequently, the remaining cells of the filter membrane faced the lower chamber was stained with Hoechst 33258 (Sigma-Aldrich). The migrated cancer cells were then visualized and counted from 5 different visual areas of 100-fold magnification under an inverted microscope.

### Soft agar clonogenicity assay

Each well (35 mm) of a six-well culture dish was coated with 2 ml bottom agar (Sigma-Aldrich) mixture (DMEM, 10% (v/v) FCS, 0.6% (w/v) agar). After the bottom layer was solidified, 2 ml top agar-medium mixture (DMEM, 10% (v/v) FCS, 0.3% (w/v) agar) containing 10^4 ^cells were added, and the dishes were incubated at 37°C for 4 weeks. Plates were stained with 0.005% Crystal Violet then the colonies were counted. The number of total colonies with a diameter = 100 μm was counted over five fields per well for a total of 15 fields in triplicate experiments.

### Immunohistochemistry

Between 1994 and 1997, 46 consecutive patients with operable head and neck cancer underwent surgery at the Department of Oral and Maxillofacial Surgery, Mackay Memorial Hospital. This research follows the tenets of the Declaration of Helsinki and all samples were obtained after informed consent from the patients. None of the subjects received radiation therapy or chemotherapy before surgery.Forty-six patients' tissue samples with different stages of oral cancer were spotted on glass slides for immunohistochemical stainings. After deparaffinization and rehydration, the tissue sections were processed with antigen retrieval by1X Trilogy diluted in H_2_O (Biogenics) and heat. The slides were immersed in 3% H_2_O_2 _for 10 minutes and washed with PBS 3 times. The tissue sections were then blocked with serum (Vestastain Elite ABC kit, Vector Laboratories, Burlingame, CA) for 30 minutes, followed by incubating with the primary antibody, anti-GRP78 (BD Transduction Laboratories™) in PBS solution at room temperature for 2 hours in a container. Tissue slides were washed with PBS and incubated with biotin-labeled secondary antibody for 30 minutes and then incubated with streptavidin-horse radish peroxidase conjugates for 30 minutes and washed with PBS 3 times. Afterwards, the tissue sections were immersed with chromogen 3-3'-diaminobenzidine plus H_2_O_2 _substrate solution (Vector^® ^DBA/Ni substrate kit, SK-4100, Vector Laboratories, Burlingame, CA) for 10 minutes. Hematoxylin was applied for counter-staining (Sigma Chemical Co., USA). Finally, the tumor sections were mounted with a cover slide with Gurr^® ^(BDH Laboratory Supplies, U.K.) and examined under a microscope. Pathologists scoring the immunohistochemistry were blinded to the clinical data. The interpretation was done in five high-power views for each slide, and 100 cells per view were counted for analysis. (-, 0-10% positive cells; +, more than 10% positive cells)

### Subcutaneous xenografts in nude mice

All the animal practices in this study were in accordance with the institutional animal welfare guideline of National Yang-Ming University, Taiwan. HNSCCs or HN-CICs subject to treatment were injected subcutaneously into BALB/c nude mice (8 weeks). Tumor volume (TV) was calculated using the following formula: TV (mm3) = (Length × Width ^2^)/2 and then analyzed using Image Pro-plus software.

### Analyses of differential gene expression profiles, mapping of human protein-protein interactions (PPIs), and functional annotation clustering

All CEL files were pre-processed using method justRMA and standardized with mean of zero and SD of 1. First, modified t-test of the 'limma' package was used for differential gene expression analysis between the control- or *GRP78-*knockdown HN-CICs, controlled for FDR < 0.05 [55]. The analysis focused on precompiled calcium, migratory [56,57]and stemness related gene lists [58,59]. Second, we further filtered out differential expression gene signatures with any inconsistent direction of regulation between any pair of control- v.s. *GRP78-*knockdown HN-CICs. Third, differentially expressed probes were mapped onto the human PPIs downloaded from the NCBI Gene Portal (HPRD, BioGrid, and BIND). PPIs would be retrieved if and only if both of the interactants were listed as of those differentially expressed. Fourth, absolute values of Pearson correlation coefficients (PCCs) of the mapped PPIs were calculated to identify cut-off threshold at 0.5 to filter out possible false-positive interactions. Finally, network topological analyses and classification of genes were performed according to methods previously published [60]. Analytical computation, hierarchical clustering and heatmap were performed and displayed using R statistical software [61]. Functional enrichment clustering of genes in the final mapped human PPIs was analyzed by DAVID (Database for Annotation Visualization and Integrated Discovery, NIH) [62].

### Transient overexpression of GRP78 in HNSCCs

To overexpress the GRP78 protein in HNSCCs, a plasmid (pCMV-GRP78; a gift from Dr. Ann-Joy Cheng, Chang Gung University, Taipei, Taiwan) which can overexpress the GRP78 in mammalian cells under CMV promoter, was introduced HNSCCs transiently by transfection. In the meanwhile, plasmids encoding green fluorescence protein were co-introduced into host cells to identify the successful transfection cells.

### Statistical analysis

The independent Student's *t*-test was used to compare the continuous variables between groups, whereas the χ^2 ^test was applied for the comparison of dichotomous variable. Statistical Package of Social Sciences software (version 13.0) (SPSS, Inc., Chicago, IL) was used for statistical Kaplan-Meier analysis. The Kaplan-Meier estimate was used for survival analysis, and the log-rank test was selected to compare the cumulative survival durations in different patient groups. The level of statistical significance was set at 0.05 for all tests.

## List of abbreviations

HNSCC: (Head and neck squamous cell carcinoma); HN-CICs: (Head and neck cancer initiating cells); GRP78: (Glucose regulated protein 78); CICs: (cancer initiating cells); CSCs: (cancer stem cells)

## Competing interests

The authors declare that they have no competing interests.

## Authors' contributions

CCY and JFL designed research. MJW, CIJ, YHY, CYH, SCL, YSC, CJL, and YGT performed research and analyzed data. CCY and JFL supervised the study and wrote the paper. All the authors have read and approved the final manuscript.

## Supplementary Material

Additional file 1 **Clustering the progressive gene expression profiles of in the HN-CICs**. The heat maps of the transcripts differentially expressed in parental HNSCCs and HNSCCs-derived HN-CICs. Red arrows indicate GRP78. Click here for file

Additional file 2 **Cancer stemness properties of ^mem^GRP78^+ ^and ^mem^GRP78^- ^HNSCCs**. **(A) **Sorted ^mem^GRP78^+ ^and ^mem^GRP78^- ^HNSCCs by flow cytometry. **(B) **Total RNA was purified from parental ^mem^GRP78^+ ^and ^mem^GRP78^- ^HNSCCs, and the expression of stemness transcript (Oct4 and Nanog) was detected by and RT-PCR analysis. **(C) **^mem^GRP78^+ ^and ^mem^GRP78^- ^cells plated onto soft agar and analyzed colony size. *In vivo *tumor growth ability of 5 × 10^5 ^**(D) **and 1 × 10^5 ^**(E) **^mem^GRP78^+ ^and ^mem^GRP78^- ^cells examined by xenotransplantation analysis. **(F) **Representative tumor growth of ^mem^GRP78^+ ^and ^mem^GRP78^- ^HNSCCs was generated in the subcutaneous space of recipient nude mice (Yellow arrows: ^mem^GRP78^+ ^HNSCCs; Red arrows: ^mem^GRP78^- ^HNSCCs). Click here for file

Additional file 3 **Depletion of GRP78 impairs *in vitro *tumorigenic properties of HNSCCs and HN-CICs**. **(A) **Down-regulation of GRP78 in HNSCCs (SAS (*left panel*) and OECM1 (*right panel*) mediated by shRNAi was validated by western blotting. (B) The percentages of ^mem^GRP78^+ ^cells in sh-GRP78 knockdown and sh-Luc HN-CICs were compared by flow cytometry analysis, respectively. (C) Differential levels of GRP78 suppression between membrane and cytosol regions in head and neck cancer initiating cells (SAS and OECM1) were examined by western blotting and flow cytometry results. (D) Single cell suspensions of sh-GRP78 and sh-Luc-expressing HNSCCs incubated with Hoechst 33342 were examined for side population by flow cytometry. (E) Tumor volume was measured after inoculation of GRP78-knockdown shRNA and sh-Luc-expressing cells. Error bars correspond to SD. Click here for file

Additional file 4 **Overexpression of GRP78 modulates expression of tumorigenic potentials of HNSSCs**. **(A) **Total proteins were prepared from control (Vector alone) and GRP78-overexpressing host cells (*left*, 293T and *right*: SAS) and analyzed by immunoblotting against anti-GRP78, or anti-GAPDH antibodies as indicated. **(B) **To elucidate the capability of migration of GRP78-overexpressing and control HNSCCs (SAS and OECM1), single cell suspension of GRP78-overexpressing or control HNSCCs were plated onto transwell and analyzed as described in Materials and Methods. Results are means ± SD of triplicate samples from three experiments. **(C) **SAS cells were transfected with GFP and/or GRP78 (GRP78^over^) overexpressing plasmids. The expression profile of GFP and ^mem^GRP78^+ ^cells were further examined by FACS analyses. Representative images were displayed (left panel). The percentages of ^mem^GRP78^+ ^cells from each experimental group were calculated using GFP positive cells as 100% successful transfection rate. Results are means ± SD of triplicate samples from three representative experiments. (*, p < 0.05; ***, p < 0.001). Click here for file

## References

[B1] Jemal A Siegel R Ward E Hao Y Xu J Murray T Thun MJ Cancer statistics, 2008 CA Cancer J Clin 2008 58 71 96 10.3322/CA.2007.0010 18287387

[B2] Haddad RI Shin DM Recent advances in head and neck cancer N Engl J Med 2008 359 1143 1154 10.1056/NEJMra0707975 18784104

[B3] Rosen JM Jordan CT The increasing complexity of the cancer stem cell paradigm Science 2009 324 1670 1673 10.1126/science.1171837 PMC2873047 19556499

[B4] Gupta PB Chaffer CL Weinberg RA Cancer stem cells: mirage or reality? Nat Med 2009 15 1010 1012 10.1038/nm0909-1010 19734877

[B5] Dean M Fojo T Bates S Tumour stem cells and drug resistance Nat Rev Cancer 2005 5 275 284 10.1038/nrc1590 15803154

[B6] Baumann M Krause M Hill R Exploring the role of cancer stem cells in radioresistance Nat Rev Cancer 2008 8 545 554 10.1038/nrc2419 18511937

[B7] Singh SK Hawkins C Clarke ID Squire JA Bayani J Hide T Henkelman RM Cusimano MD Dirks PB Identification of human brain tumour initiating cells Nature 2004 432 396 401 10.1038/nature03128 15549107

[B8] Al-Hajj M Wicha MS Benito-Hernandez A Morrison SJ Clarke MF Prospective identification of tumorigenic breast cancer cells Proc Natl Acad Sci USA 2003 100 3983 3988 10.1073/pnas.0530291100 PMC153034 12629218

[B9] Ricci-Vitiani L Lombardi DG Pilozzi E Biffoni M Todaro M Peschle C De Maria R Identification and expansion of human colon-cancer-initiating cells Nature 2007 445 111 115 10.1038/nature05384 17122771

[B10] Lawson DA Zong Y Memarzadeh S Xin L Huang J Witte ON Basal epithelial stem cells are efficient targets for prostate cancer initiation Proc Natl Acad Sci USA 2010 107 2610 2615 10.1073/pnas.0913873107 PMC2823887 20133806

[B11] Kim CF Jackson EL Woolfenden AE Lawrence S Babar I Vogel S Crowley D Bronson RT Jacks T Identification of bronchioalveolar stem cells in normal lung and lung cancer Cell 2005 121 823 835 10.1016/j.cell.2005.03.032 15960971

[B12] Visvader JE Lindeman GJ Cancer stem cells in solid tumours: accumulating evidence and unresolved questions Nat Rev Cancer 2008 8 755 768 10.1038/nrc2499 18784658

[B13] Chiou SH Yu CC Huang CY Lin SC Liu CJ Tsai TH Chou SH Chien CS Ku HH Lo JF Positive correlations of Oct-4 and Nanog in oral cancer stem-like cells and high-grade oral squamous cell carcinoma Clin Cancer Res 2008 14 4085 4095 10.1158/1078-0432.CCR-07-4404 18593985

[B14] Wang M Ye R Barron E Baumeister P Mao C Luo S Fu Y Luo B Dubeau L Hinton DR Lee AS Essential role of the unfolded protein response regulator GRP78/BiP in protection from neuronal apoptosis Cell Death Differ 2010 17 488 498 10.1038/cdd.2009.144 PMC2822118 19816510

[B15] Gonzalez-Gronow M Selim MA Papalas J Pizzo SV GRP78: a multifunctional receptor on the cell surface Antioxid Redox Signal 2009 11 2299 2306 10.1089/ARS.2009.2568 19331544

[B16] Luo S Mao C Lee B Lee AS GRP78/BiP Is Required for Cell Proliferation and Protecting the Inner Cell Mass from Apoptosis during Early Mouse Embryonic Development Mol Cell Biol 2006 26 5688 5697 10.1128/MCB.00779-06 PMC1592753 16847323

[B17] Lee AS GRP78 induction in cancer: therapeutic and prognostic implications Cancer Res 2007 67 3496 3499 10.1158/0008-5472.CAN-07-0325 17440054

[B18] Uramoto H Sugio K Oyama T Nakata S Ono K Yoshimastu T Morita M Yasumoto K Expression of endoplasmic reticulum molecular chaperone Grp78 in human lung cancer and its clinical significance Lung Cancer 2005 49 55 62 10.1016/j.lungcan.2004.12.011 15949590

[B19] Dong D Ni M Li J Xiong S Ye W Virrey JJ Mao C Ye R Wang M Pen L Critical role of the stress chaperone GRP78/BiP in tumor proliferation, survival, and tumor angiogenesis in transgene-induced mammary tumor development Cancer Res 2008 68 498 505 10.1158/0008-5472.CAN-07-2950 18199545

[B20] Dong D Ko B Baumeister P Swenson S Costa F Markland F Stiles C Patterson JB Bates SE Lee AS Vascular targeting and antiangiogenesis agents induce drug resistance effector GRP78 within the tumor microenvironment Cancer Res 2005 65 5785 5791 10.1158/0008-5472.CAN-05-0754 15994954

[B21] Lee E Nichols P Spicer D Groshen S Yu MC Lee AS GRP78 as a novel predictor of responsiveness to chemotherapy in breast cancer Cancer Res 2006 66 7849 7853 10.1158/0008-5472.CAN-06-1660 16912156

[B22] Chiu C-C Lin C-Y Lee L-Y Chen Y-J Kuo T-F Chang JT-C Liao C-T Wang H-M Yen T-C Shen C-R Glucose-regulated protein 78 regulates multiple malignant phenotypes in head and neck cancer and may serve as a molecular target of therapeutic intervention Molecular Cancer Therapeutics 2008 7 2788 2797 10.1158/1535-7163.MCT-08-0172 18790759

[B23] Bartkowiak K Effenberger KE Harder S Andreas A Buck F Peter-Katalinic J Pantel K Brandt BH Discovery of a novel unfolded protein response phenotype of cancer stem/progenitor cells from the bone marrow of breast cancer patients J Proteome Res 2010 9 3158 3168 10.1021/pr100039d 20423148

[B24] Misra UK Pizzo SV Modulation of the unfolded protein response in prostate cancer cells by antibody-directed against the carboxyl-terminal domain of GRP78 Apoptosis 2010 15 173 182 10.1007/s10495-009-0430-y 20091233

[B25] Chen YC Chen YW Hsu HS Tseng LM Huang PI Lu KH Chen DT Tai LK Yung MC Chang SC Aldehyde dehydrogenase 1 is a putative marker for cancer stem cells in head and neck squamous cancer Biochem Biophys Res Commun 2009 385 307 313 10.1016/j.bbrc.2009.05.048 19450560

[B26] Clay MR Tabor M Owen JH Carey TE Bradford CR Wolf GT Wicha MS Prince ME Single-marker identification of head and neck squamous cell carcinoma cancer stem cells with aldehyde dehydrogenase Head Neck 2010 32 1195 1201 10.1002/hed.21315 PMC2991066 20073073

[B27] Strizzi L Abbott DE Salomon DS Hendrix MJC Potential for Cripto-1 in defining stem cell-like characteristics in human malignant melanoma Cell cycle 2008 7 1931 1935 10.4161/cc.7.13.6236 PMC2556902 18604175

[B28] Liu G Yuan X Zeng Z Tunici P Ng H Abdulkadir IR Lu L Irvin D Black KL Yu JS Analysis of gene expression and chemoresistance of CD133+ cancer stem cells in glioblastoma Mol Cancer 2006 5 67 10.1186/1476-4598-5-67 PMC1697823 17140455

[B29] Wu C Alman BA Side population cells in human cancers Cancer Lett 2008 268 1 9 10.1016/j.canlet.2008.03.048 18487012

[B30] Lin CY Chen WH Liao CT Chen IH Chiu CC Wang HM Yen TC Lee LY Chang JT Cheng AJ Positive association of glucose-regulated protein 78 during oral cancer progression and the prognostic value in oral precancerous lesions Head Neck 2010 32 1028 1039 10.1002/hed.21287 19953611

[B31] Dubrovska A Kim S Salamone RJ Walker JR Maira SM Garcia-Echeverria C Schultz PG Reddy VA The role of PTEN/Akt/PI3K signaling in the maintenance and viability of prostate cancer stem-like cell populations Proc Natl Acad Sci USA 2009 106 268 273 10.1073/pnas.0810956106 PMC2629188 19116269

[B32] Tabu K Kimura T Sasai K Wang L Bizen N Nishihara H Taga T Tanaka S Analysis of an alternative human CD133 promoter reveals the implication of Ras/ERK pathway in tumor stem-like hallmarks Mol Cancer 2010 9 39 10.1186/1476-4598-9-39 PMC2836276 20167130

[B33] Mousa SA Sudha T Dyskin E Dier U Gallati C Hanko C Chittur SV Rebbaa A Stress resistant human embryonic stem cells as a potential source for the identification of novel cancer stem cell markers Cancer Lett 2010 289 208 216 10.1016/j.canlet.2009.08.018 19733430

[B34] Kang J Shakya A Tantin D Stem cells, stress, metabolism and cancer: a drama in two Octs Trends Biochem Sci 2009 34 491 499 10.1016/j.tibs.2009.06.003 19733480

[B35] Philippova M Ivanov D Joshi MB Kyriakakis E Rupp K Afonyushkin T Bochkov V Erne P Resink TJ Identification of proteins associating with glycosylphosphatidylinositol- anchored T-cadherin on the surface of vascular endothelial cells: role for Grp78/BiP in T-cadherin-dependent cell survival Mol Cell Biol 2008 28 4004 4017 10.1128/MCB.00157-08 PMC2423122 18411300

[B36] Baumeister P Dong D Fu Y Lee AS Transcriptional induction of GRP78/BiP by histone deacetylase inhibitors and resistance to histone deacetylase inhibitor-induced apoptosis Mol Cancer Ther 2009 8 1086 1094 10.1158/1535-7163.MCT-08-1166 PMC2889001 19417144

[B37] Miyake H Hara I Arakawa S Kamidono S Stress protein GRP78 prevents apoptosis induced by calcium ionophore, ionomycin, but not by glycosylation inhibitor, tunicamycin, in human prostate cancer cells J Cell Biochem 2000 77 396 408 10.1002/(sici)1097-4644(20000601)77:3<396::aid-jcb5>3.0.co;2-5 10760948

[B38] Lee HK Xiang C Cazacu S Finniss S Kazimirsky G Lemke N Lehman NL Rempel SA Mikkelsen T Brodie C GRP78 is overexpressed in glioblastomas and regulates glioma cell growth and apoptosis Neuro Oncol 2008 10 236 243 10.1215/15228517-2008-006 PMC2563046 18403493

[B39] Misra UK Deedwania R Pizzo SV Activation and cross-talk between Akt, NF-kappaB, and unfolded protein response signaling in 1-LN prostate cancer cells consequent to ligation of cell surface-associated GRP78 J Biol Chem 2006 281 13694 13707 10.1074/jbc.M511694200 16543232

[B40] Lin Y Wang Z Liu L Chen L Akt is the downstream target of GRP78 in mediating cisplatin resistance in ER stress-tolerant human lung cancer cells Lung Cancer 2010 in press 10.1016/j.lungcan.2010.06.004 20599289

[B41] Kelber JA Panopoulos AD Shani G Booker EC Belmonte JC Vale WW Gray PC Blockade of Cripto binding to cell surface GRP78 inhibits oncogenic Cripto signaling via MAPK/PI3K and Smad2/3 pathways Oncogene 2009 28 2324 2336 10.1038/onc.2009.97 PMC2749668 19421146

[B42] Mazumdar J Dondeti V Simon MC Hypoxia-inducible factors in stem cells and cancer J Cell Mol Med 2009 13 4319 4328 10.1111/j.1582-4934.2009.00963.x PMC2874971 19900215

[B43] Rankin EB Giaccia AJ The role of hypoxia-inducible factors in tumorigenesis Cell Death Differ 2008 15 678 685 10.1038/cdd.2008.21 PMC3050610 18259193

[B44] Das B Tsuchida R Malkin D Koren G Baruchel S Yeger H Hypoxia enhances tumor stemness by increasing the invasive and tumorigenic side population fraction Stem Cells 2008 26 1818 1830 10.1634/stemcells.2007-0724 18467664

[B45] Heddleston JM Li Z Lathia JD Bao S Hjelmeland AB Rich JN Hypoxia inducible factors in cancer stem cells Br J Cancer 2010 102 789 795 10.1038/sj.bjc.6605551 PMC2833246 20104230

[B46] Soeda A Park M Lee D Mintz A Androutsellis-Theotokis A McKay RD Engh J Iwama T Kunisada T Kassam AB Hypoxia promotes expansion of the CD133-positive glioma stem cells through activation of HIF-1alpha Oncogene 2009 28 3949 3959 10.1038/onc.2009.252 19718046

[B47] McCord AM Jamal M Shankavaram UT Lang FF Camphausen K Tofilon PJ Physiologic oxygen concentration enhances the stem-like properties of CD133+ human glioblastoma cells in vitro Mol Cancer Res 2009 7 489 497 10.1158/1541-7786.MCR-08-0360 PMC6290460 19372578

[B48] Covello KL Kehler J Yu H Gordan JD Arsham AM Hu CJ Labosky PA Simon MC Keith B HIF-2alpha regulates Oct-4: effects of hypoxia on stem cell function, embryonic development, and tumor growth Genes Dev 2006 20 557 570 10.1101/gad.1399906 PMC1410808 16510872

[B49] Wouters BG Koritzinsky M Hypoxia signalling through mTOR and the unfolded protein response in cancer Nat Rev Cancer 2008 8 851 864 10.1038/nrc2501 18846101

[B50] Bartkowiak K Effenberger KE Harder S Andreas A Buck F Pantel K Peter-Katalinic J Brandt B Discovery of a novel unfolded protein response phenotype of cancer stem/progenitor cells from the bone marrow of breast cancer patients J Proteome Res 2010 10.1021/pr100039d 20423148

[B51] Ostergaard L Simonsen U Eskildsen-Helmond Y Vorum H Uldbjerg N Honore B Mulvany MJ Proteomics reveals lowering oxygen alters cytoskeletal and endoplasmatic stress proteins in human endothelial cells Proteomics 2009 9 4457 4467 10.1002/pmic.200800130 19670369

[B52] Arnaudeau S Arboit P Bischof P Shin-ya K Tomida A Tsuruo T Irion O Cohen M Glucose-regulated protein 78: a new partner of p53 in trophoblast Proteomics 2009 9 5316 5327 10.1002/pmic.200800865 20017148

[B53] Lin T Chao C Saito S Mazur SJ Murphy ME Appella E Xu Y p53 induces differentiation of mouse embryonic stem cells by suppressing Nanog expression Nat Cell Biol 2005 7 165 171 10.1038/ncb1211 15619621

[B54] Fu Y Wey S Wang M Ye R Liao CP Roy-Burman P Lee AS Pten null prostate tumorigenesis and AKT activation are blocked by targeted knockout of ER chaperone GRP78/BiP in prostate epithelium Proc Natl Acad Sci USA 2008 105 19444 19449 10.1073/pnas.0807691105 PMC2614780 19033462

[B55] Strimmer K A unified approach to false discovery rate estimation BMC Bioinformatics 2008 9 303 10.1186/1471-2105-9-303 PMC2475539 18613966

[B56] Simpson KJ Selfors LM Bui J Reynolds A Leake D Khvorova A Brugge JS Identification of genes that regulate epithelial cell migration using an siRNA screening approach Nat Cell Biol 2008 10 1027 1038 10.1038/ncb1762 19160483

[B57] Zaidel-Bar R Itzkovitz S Ma'ayan A Iyengar R Geiger B Functional atlas of the integrin adhesome Nat Cell Biol 2007 9 858 867 10.1038/ncb0807-858 PMC2735470 17671451

[B58] Muller FJ Laurent LC Kostka D Ulitsky I Williams R Lu C Park IH Rao MS Shamir R Schwartz PH Regulatory networks define phenotypic classes of human stem cell lines Nature 2008 455 401 405 10.1038/nature07213 PMC2637443 18724358

[B59] Ben-Porath I Thomson MW Carey VJ Ge R Bell GW Regev A Weinberg RA An embryonic stem cell-like gene expression signature in poorly differentiated aggressive human tumors Nat Genet 2008 40 499 507 10.1038/ng.127 PMC2912221 18443585

[B60] Yu YH Kuo HK Chang KW The evolving transcriptome of head and neck squamous cell carcinoma: a systematic review PLoS One 2008 3 e3215 10.1371/journal.pone.0003215 PMC2533097 18791647

[B61] Gentleman RC Carey VJ Bates DM Bolstad B Dettling M Dudoit S Ellis B Gautier L Ge Y Gentry J Bioconductor: open software development for computational biology and bioinformatics Genome Biol 2004 5 R80 10.1186/gb-2004-5-10-r80 PMC545600 15461798

[B62] Huang da W Sherman BT Lempicki RA Systematic and integrative analysis of large gene lists using DAVID bioinformatics resources Nat Protoc 2009 4 44 57 10.1038/nprot.2008.211 19131956

