# Supplementary figures and images for: Elimination of head and neck cancer initiating cells through targeting glucose regulated protein78 signaling
Source: Mol Cancer. 2010 Oct 27;9:283. doi: 10.1186/1476-4598-9-283 (PMC2987982; doi:10.1186/1476-4598-9-283)

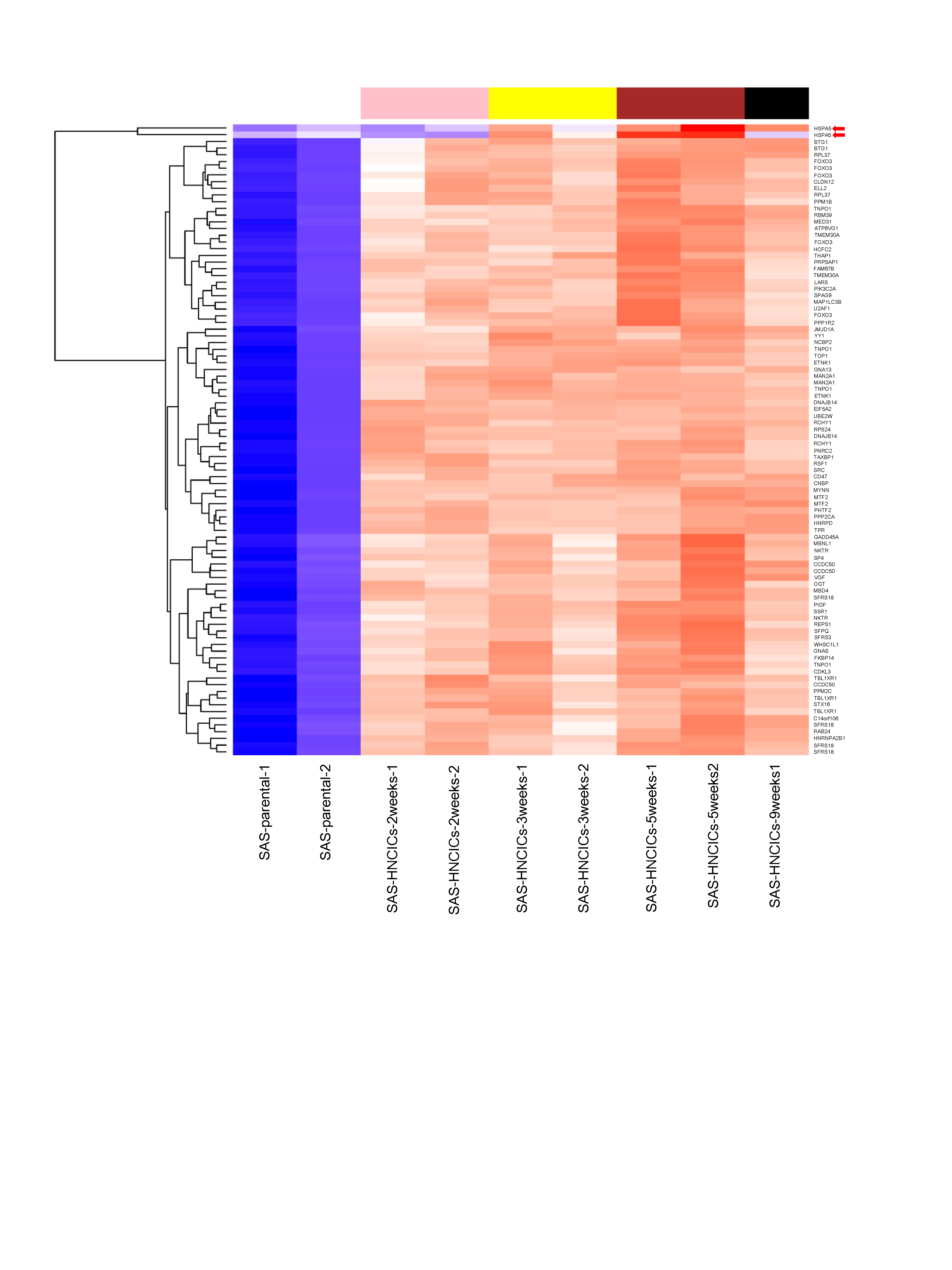

Supplement: Additional file 1 — Clustering the progressive gene expression profiles of in the HN-CICs. The heat maps of the transcripts differentially expressed in parental HNSCCs and HNSCCs-derived HN-CICs. Red arrows indicate GRP78. [file 1476-4598-9-283-S1.TIFF]

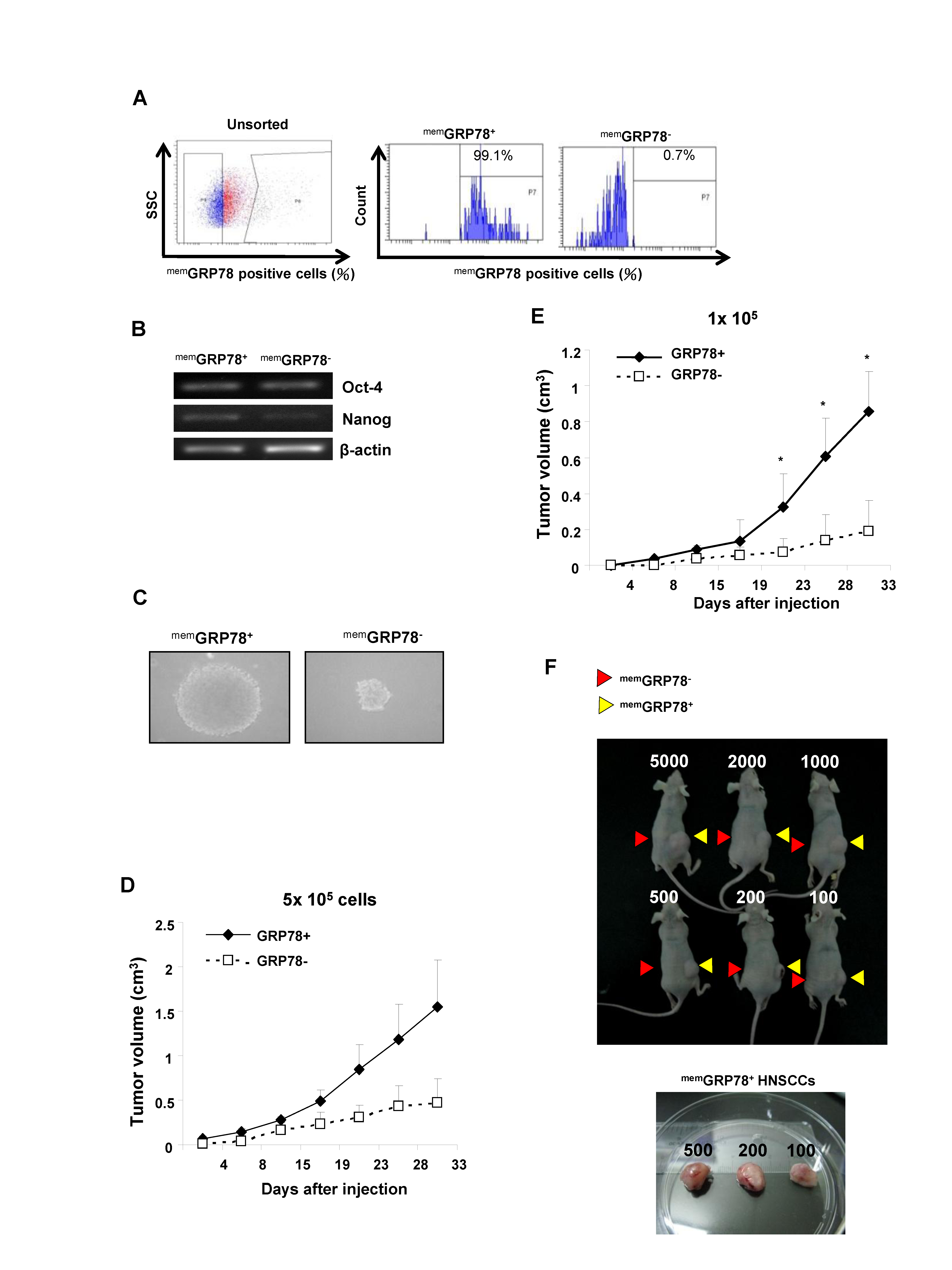

Supplement: Additional file 2 — Cancer stemness properties of memGRP78+ and memGRP78- HNSCCs. (A) Sorted memGRP78+ and memGRP78- HNSCCs by flow cytometry. (B) Total RNA was purified from parental memGRP78+ and memGRP78- HNSCCs, and the expression of stemness transcript (Oct4 and Nanog) was detected by and RT-PCR analysis. (C) memGRP78+ and memGRP78- cells plated onto soft agar and analyzed colony size. In vivo tumor growth ability of 5 × 10^5^ (D) and 1 × 10^5^ (E) memGRP78+ and memGRP78- cells examined by xenotransplantation analysis. (F) Representative tumor growth of memGRP78+ and memGRP78- HNSCCs was generated in the subcutaneous space of recipient nude mice (Yellow arrows: memGRP78+ HNSCCs; Red arrows: memGRP78- HNSCCs). [file 1476-4598-9-283-S2.TIFF]

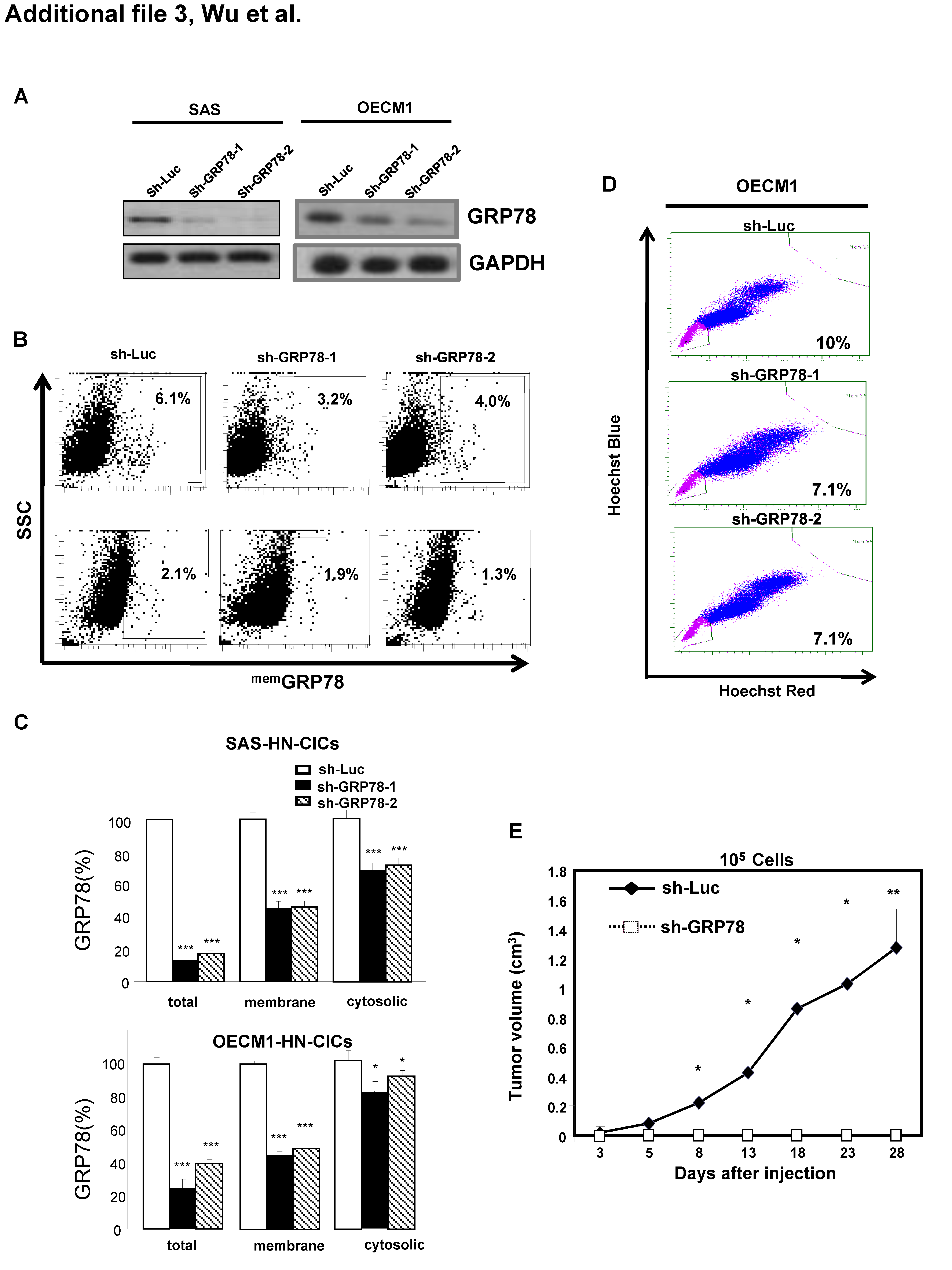

Supplement: Additional file 3 — Depletion of GRP78 impairs in vitro tumorigenic properties of HNSCCs and HN-CICs. (A) Down-regulation of GRP78 in HNSCCs (SAS (left panel) and OECM1 (right panel) mediated by shRNAi was validated by western blotting. (B) The percentages of memGRP78+ cells in sh-GRP78 knockdown and sh-Luc HN-CICs were compared by flow cytometry analysis, respectively. (C) Differential levels of GRP78 suppression between membrane and cytosol regions in head and neck cancer initiating cells (SAS and OECM1) were examined by western blotting and flow cytometry results. (D) Single cell suspensions of sh-GRP78 and sh-Luc-expressing HNSCCs incubated with Hoechst 33342 were examined for side population by flow cytometry. (E) Tumor volume was measured after inoculation of GRP78-knockdown shRNA and sh-Luc-expressing cells. Error bars correspond to SD. [file 1476-4598-9-283-S3.TIFF]

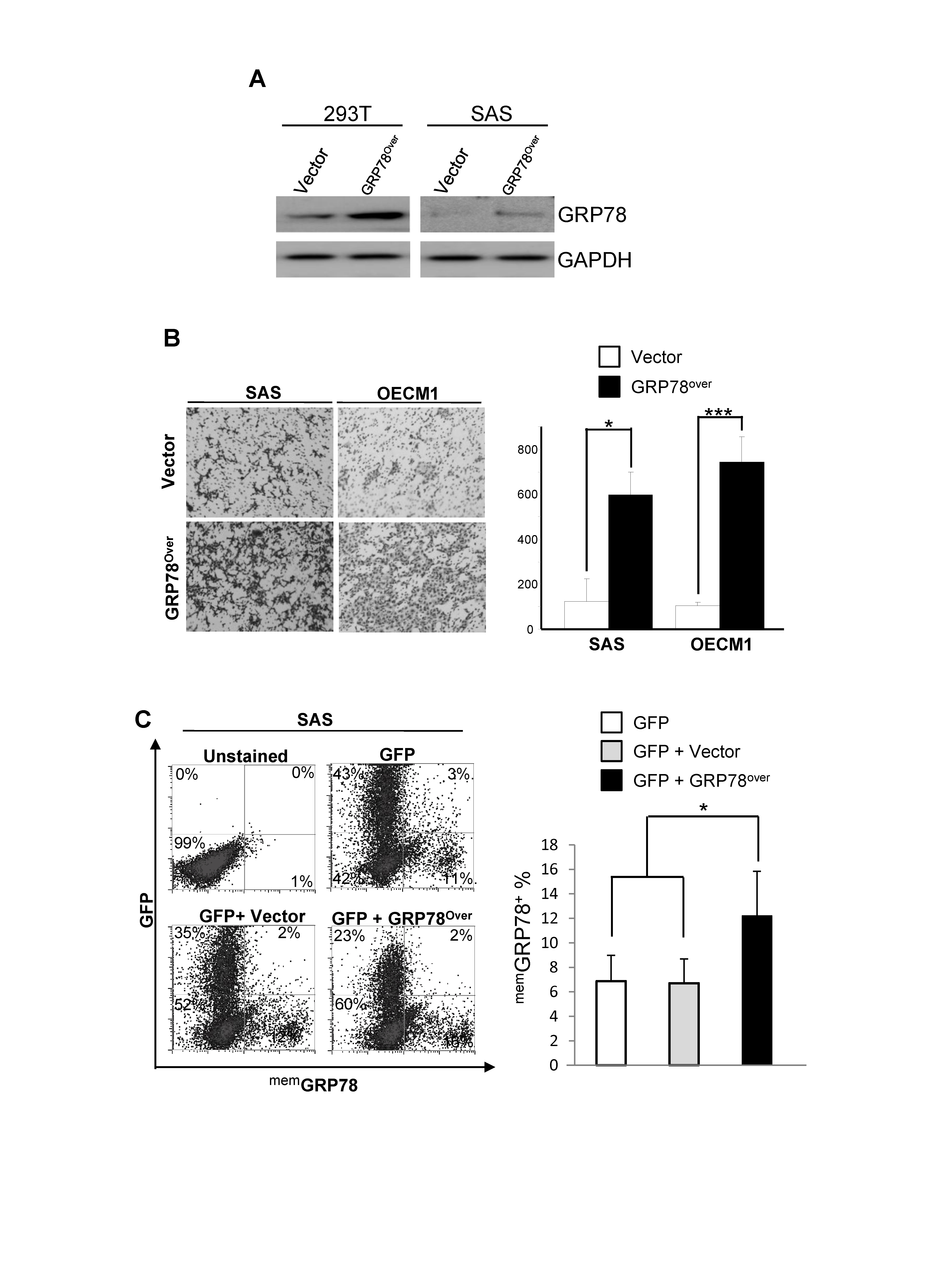

Supplement: Additional file 4 — Overexpression of GRP78 modulates expression of tumorigenic potentials of HNSSCs. (A) Total proteins were prepared from control (Vector alone) and GRP78-overexpressing host cells (left, 293T and right: SAS) and analyzed by immunoblotting against anti-GRP78, or anti-GAPDH antibodies as indicated. (B) To elucidate the capability of migration of GRP78-overexpressing and control HNSCCs (SAS and OECM1), single cell suspension of GRP78-overexpressing or control HNSCCs were plated onto transwell and analyzed as described in Materials and Methods. Results are means ± SD of triplicate samples from three experiments. (C) SAS cells were transfected with GFP and/or GRP78 (GRP78over) overexpressing plasmids. The expression profile of GFP and memGRP78+ cells were further examined by FACS analyses. Representative images were displayed (left panel). The percentages of memGRP78+ cells from each experimental group were calculated using GFP positive cells as 100% successful transfection rate. Results are means ± SD of triplicate samples from three representative experiments. (*, p < 0.05; ***, p < 0.001). [file 1476-4598-9-283-S4.TIFF]
